# Adropin deficiency worsens HFD-induced metabolic defects

**DOI:** 10.1038/cddis.2017.362

**Published:** 2017-08-24

**Authors:** Shi Chen, Kai Zeng, Qi-cai Liu, Zheng Guo, Sheng Zhang, Xiao-rong Chen, Jian-hua Lin, Jun-ping Wen, Cheng-fei Zhao, Xin-hua Lin, Feng Gao

**Affiliations:** 1 Department of Hepatobiliary Surgery, Fujian Provincial Hospital, Fujian Medical University, Fuzhou, China; 2 Department of Anesthesiology, 1st Affiliated Hospital, Fujian Medical University, Fuzhou, China; 3 Department of Laboratory Medicine, 1st Affiliated Hospital, Fujian Medical University, Fuzhou, China; 4 Department of Bioinformatics, Fujian Medical University, Fuzhou, China; 5 Department of Pathology, 1st Affiliated Hospital, Fujian Medical University, Fuzhou, China; 6 Department of Radiology, 1st Affiliated Hospital, Fujian Medical University, Fuzhou, China; 7 Department of Central Laboratory, 1st Affiliated Hospital, Fuzhou, China; 8 Department of Endocrinology, Fujian Provincial Hospital, Fuzhou, China; 9 Department of Pharmaceutical Analysis, Putian University, Putian, China; 10 Department of Pharmaceutical Analysis, Fujian Medical University, Fuzhou, China

## Abstract

The limited efficacy of current treatment methods and increased type 2 diabetes mellitus (T2DM) incidence constitute an incentive for investigating how metabolic homeostasis is maintained, to improve treatment efficacy and identify novel treatment methods. We analyzed a three-generation family of Chinese origin with the common feature of T2DM attacks and fatty pancreas (FP), alongside 19 unrelated patients with FP and 58 cases with T2DM for genetic variations in *Enho*, serum adropin, and relative T_reg_ amounts. Functional studies with adropin knockout (AdrKO) in C57BL/6J mice were also performed. It showed serum adropin levels were significantly lower in FP and T2DM patients than in healthy subjects; relative T_reg_ amounts were also significantly decreased in FP and T2DM patients, and positively associated with adropin (*r*=0.7220, *P*=0.0001). Sequencing revealed that the patients shared a Cys56Trp mutation in *Enho*. *In vivo*, adropin-deficiency was associated with increased severity of glucose homeostasis impairment and fat metabolism disorder. AdrKO mice exhibited reduced endothelial nitric oxide synthase (eNOS) phosphorylation (Ser1177), impaired glycosphingolipid biosynthesis, adipocytes infiltrating, and loss of T_reg_, and developed FP and T2DM. Adropin-deficiency contributed to loss of T_reg_ and the development of FP disease and T2DM.

Obesity arises from a sustained positive energy balance that triggers a pro-inflammatory response, a key contributor to metabolic diseases such as T2DM (type 2 diabetes mellitus) and pancreatic steatosis.^1^ Specific metabolites can modulate the functional nature and inflammatory phenotype of immune cells. In obesity, the expanding adipose tissue attracts immune cells, creating an inflammatory environment within this fatty acid storage organ.^2^ Inflammatory mediators, such as TNF-*α* and IL-1, are induced by saturated fatty acids, and disrupt insulin signaling and metabolic switch in their function. Ectopic fat can also affect pancreatic *β*-cell function, thereby contributing to insulin resistance.^3, 4, 5^

In the obese state, the storage capacity of adipose tissue is exceeded. Free fatty acids (FFAs) ‘spill over’ and accumulate in metabolic tissues such as the skeletal muscle, liver, and pancreas, causing lipotoxicity. Excess FFAs in turn activate inflammatory pathways and impair normal cell signaling within immune cells and adipose tissue, as well as the liver and muscle, causing cellular dysfunction.^6^ Consequently, metabolic disorders such as insulin resistance and type 2 diabetes can develop. Similar to the liver and skeletal muscle, the pancreas is a metabolic organ negatively impacted by obesity-induced lipotoxicity and glucotoxicity.^7^ Indeed, obesity-associated insulin resistance increases the metabolic demand on *β*-cells.^8^ Eventually, these cells are unable to continue the compensatory mechanism; hyperglycemia ensues, driven by the elevated FFA levels. The combined deleterious effects of glucotoxicity and lipotoxicity, referred to as glucolipotoxicity, eventually causes *β*-cell failure characteristic of T2DM.^9^ Chronic hyperglycemia as found in obesity-induced insulin resistance promotes the development of glucotoxicity.^10^ Several peptide hormones secreted by the endocrine pancreas, gut, adipocytes, and liver modulate insulin activity to maintain glucose homeostasis and fat amounts; these hormones are considered promising leads in the development of therapies against T2DM and fatty liver or pancreas disease.^11, 12^

Adropin is a peptide hormone that was originally described as a secreted peptide, with residues 1-33 encoding a secretory signal peptide sequence. It plays a role in energy homeostasis as well as glucose and fatty acid metabolism. This protein is encoded by the *Enho* (Energy Homeostasis Associated) gene, which is expressed primarily in the liver, pancreas, and central nervous system. How adropin secretion is secreted remains controversial. It was shown that adropin is mainly regulated by miRNAs, along with the only gene responsible for nonshivering thermogenesis (mitochondrial uncoupling protein 1, or UCP1) in brown adipose tissue.^13^ Moreover, adropin-deficiency exhibits loss of T_reg_ and results in autoimmune diseases.^13^

T_reg_ are involved in controlling the inflammatory state of adipose tissue, and thus insulin sensitivity.^14^ Although visceral adipose tissue invasion by proinflammatory macrophages is considered a key event driving adipose-tissue inflammation and insulin resistance, little is known about the roles of T_reg_ in these processes^15, 16^ and the driver of T_reg_.^17^ Expectedly, *in vivo*, T_reg_ responses are necessary for complete restoration of insulin sensitivity and dyslipidemia.^16, 17^ Adropin is involved in the mechanism of increased adiposity, insulin resistance, and glucose and lipid metabolism.^18^ More interestingly, we found that almost all patients with pancreatic steatosis were diagnosed with diabetes; in addition, *Enho* mutations were found in a three-generation family of Chinese origin with the common feature of T2DM attacks and FP. Therefore, adropin may play a role in the pathogeneses of FP and T2DM.

## Results

### Clinical characteristics

**Figure 1:**
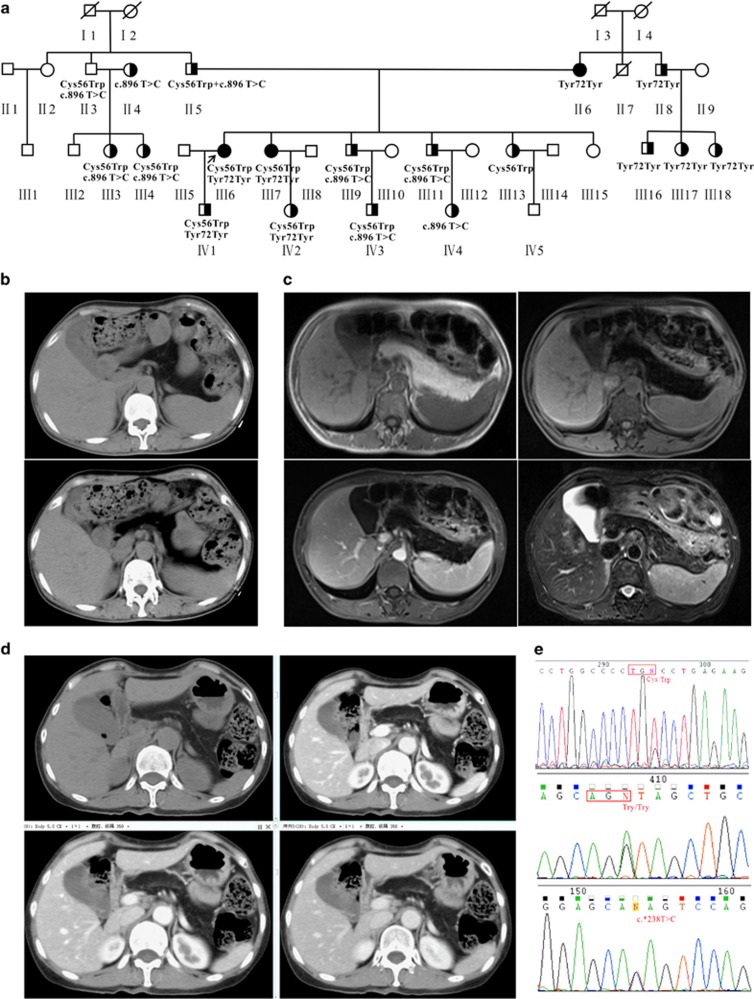

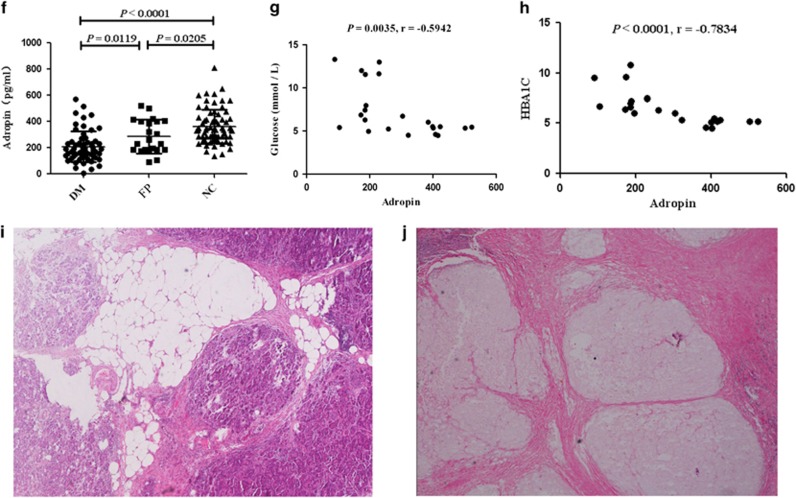
Identification of *Enho* mutations in fatty pancreas and diabetes. (**a**) The pedigree of the family affected by fatty pancreas and diabetes, fatty pancreas patients (▪●), type II diabetes mellitus patients (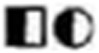) and their family normal members (○□), proband (↗). (**b**) Computed tomography (CT) revealed total homogenous replacement of the pancreas by fat (top), fat-suppression showed pancreatic signal reduction and decreased pancreatic parenchyma (bottom). (**c**) MRI T2WI and T1WI showed fatty tissues were seen within the region of the pancreas from the proband (III6). Left top: T1WI, Left bottom: T1WI fat-suppression (enhanced), Right top: T1WI fat-suppression, Right bottom: T2WI fat-suppression. (**d**) Patient III7, the sister of the proband: CT and CT enhanced scan showed pancreas morphology remained visible and pancreatic duct resulted in a fishbone like change, normal pancreatic tissue was substituted by adipose tissue. (**e**) p.Cys56Trp, p.Tyr72Tyr, and c.*238T>C mutations which were validated by Sanger sequencing. (**f**) The medium levels of serum adropin before therapy in the patients with fatty pancreas and diabetes and that of the healthy subjects. (**g**) Serum adropin inversely associated with glucose. (**h**) Serum adropin inversely associated with HbA1c. (**i**) Pancreatic steatosis is histologically characterized by an increased number of adipocytes or expansion of existing adipocyte size (III7). (**j**) Fibrosis and fat in intralobular locations in the pancreatic tissue (III6)

A 53-year-old female (the proband, III6) (Figure 1a) presented with chief complaints of foul smelling stools, with a high frequency of 3–4 times a day for the last 10 years. Stools were copious in amount and difficult to flush, floating in the pan. She was hospitalized at the age of 10 years, and diagnosed with pancreatic insufficiency; diabetes was diagnosed when she was 23. Pancreatic enzyme supplements were started, and her diarrhea improved. There was no history of jaundice/pruritus/pale stool/osmotic symptoms or any signs suggestive of pancreatitis/pancreatic cancer. Computed tomography (CT) of the abdomen revealed total homogenous replacement of the pancreas by fat (Figure 1b). MRI T2WI and T1WI showed fatty tissue within the region of the pancreas. There was almost no normal pancreatic parenchyma, and the area was completely filled with adipose tissue (Figure 1c).

Because most of her family members suffered from diabetes or/and fatty pancreas (FP), a detailed investigation was carried out to further assess the relationship between FP and diabetes. Family history was notable for the appearance of similar symptoms in multiple members of this family across three generations, with the common feature of diabetes attacks. The pedigree of this family contained 32 members, including 18 subjects with diabetes or FP phenotype and 14 without phenotype (Figure 1a). The family members with diabetes had a similar age of onset (20–32 years old). Patient III7, the proband’s sister, a 60-year-old female, suffered from pancreatic insufficiency at the age of 16, and was diagnosed with diabetes at 30 years old. CT and enhanced CT scan showed the pancreatic duct had a fishbone like change, with normal pancreatic tissue substituted by adipose tissue (Figure 1d). Their mother (II: 6) was 85 years old, with diabetes and FP disease, as well as heart disease. The proband’s uncle (II: 7) died from stroke at the age of 52 years.

Genetic analysis of *Enho* (ENSMUSG00000028445) from the proband showed a heterozygous mutation (c.168T>G), the well-known p.Cys56Trp, which originated from the father (II5). This mutation was confirmed by Sanger sequencing in every affected member of the family who consented to genetic analysis (II3, 5 and III3, 4, 6, 7, 9, 11 and IV1, 2, 3), suggesting a high penetration of this mutation (Figure 1e). Moreover, c.216 C>T heterozygous synonymous mutation (ENST00000399775.2: p.Tyr72Tyr) (Figure 1e) was also found in the family (II3, 5 and III3, 4, 6, 7, 9, 11 and IV1, 2, 3), and originated from the mother (II6); the mutation was located at the predicted tyrosine phosphorylation site.^13^ Both mutations were not detected in the other five healthy members (II9 and III2, 5, 8, 15) without the diagnostic feature of diabetes or FP. The other nine patients harbored c.*238T>C mutation at the 3′ UTR of *Enho* (Figure1e). Moreover, p.Cys56Trp was also found in six unrelated patients with FP and eight cases with T2DM, and p.Tyr72Tyr in six unrelated patients with FP and 12 cases with T2DM. However, none of the mutations were found in control participants.

### Loss of adropin and T_reg_ in patients with FP and T2DM

Medium levels of serum adropin before therapy were significantly lower in patients with FP than in healthy subjects (*n*=22, 244.50 pg/ml (89.00–523.00 pg/ml) and *n*=72, 336.88 pg/ml (136.20–811.75 pg/ml), respectively; *P*=0.0205). In addition, lower levels were also found in patients with T2DM compared with the normal control group (*n*=58, 178.13 pg/ml; 7.15–569.20 pg/ml, *P*<0.0001) (Figure 1f). Moreover, serum adropin levels were lower in the T2DM group than FP patients (*P*=0.0119, T2DM *versus* FP). More excitingly, serum adropin was inversely associated with glucose (*r*=−0.5942, *P*=0.0035) (Figure 1g) and HbA1c (*r*=−0.7834, *P*<0.0001) (Figure 1h).

Unlike non-alcoholic fatty liver disease, where triglyceride accumulation is mainly intracellular, pancreatic steatosis is histologically characterized by an increased number of adipocytes, a size expansion of existing adipocytes (Figure 1i), fibrosis and fat in intra-lobular locations (Figure 1j). Interestingly, almost all patients with FP were diagnosed with T2DM.

**Figure 2:**
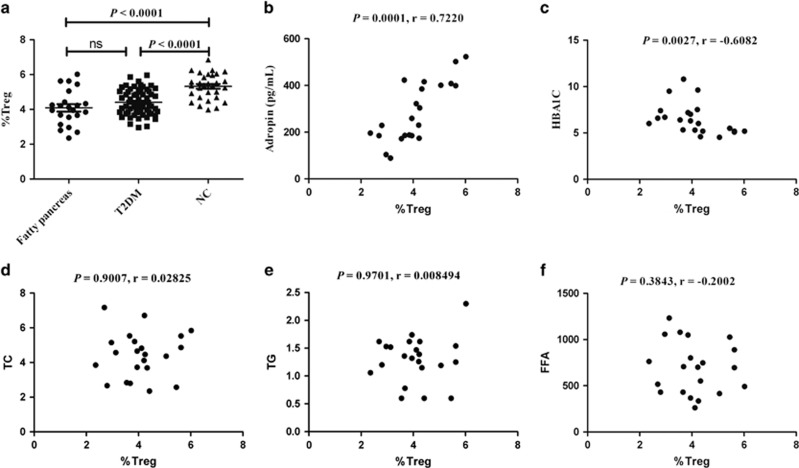
Loss of adropin and T_reg_ cells in the patients with FP and T2DM. (**a**) The relative numbers of T_reg_ cells were significantly decreased in patients with FP and T2DM. (**b**) The relative numbers of T_reg_ cells were positively associated with adropin. (**c**) The relative numbers of T_reg_ cells were inversely associated with HbA1c. (**d**) The relative numbers of T_reg_ cells was not relative to total cholesterol (TC). (**e**) The relative numbers of T_reg_ cells was not relative to total glyceride (TG). (**f**) The relative numbers of T_reg_ cells was not relative to free fatty acids (FFA)

Furthermore, relative T_reg_ amounts were significantly decreased in patients with FP and T2DM (*P*<0.0001, *P*<0.0001, *versus* normal control) (Figure 2a), positively associated with adropin levels (*r*=0.7220, *P*=0.0001) (Figure 2b), and inversely associated with hemoglobin A1C (HbA1c) (*r*=−0.6082, *P*=0.0027) (Figure 2c). Surprisingly, T_reg_ amounts were not correlated with total cholesterol (*r*=0.02825, *P*=0.9007) (Figure 2d), total glyceride (TG) (*r*=0.008494, *P*=0.9701) (Figure 2e), and FFA (*r*=−0.2002, *P*=0.3843) (Figure 2f).

### Pathogenesis of fatty pancreas disease and diabetes in AdrKO mice

**Figure 3:**
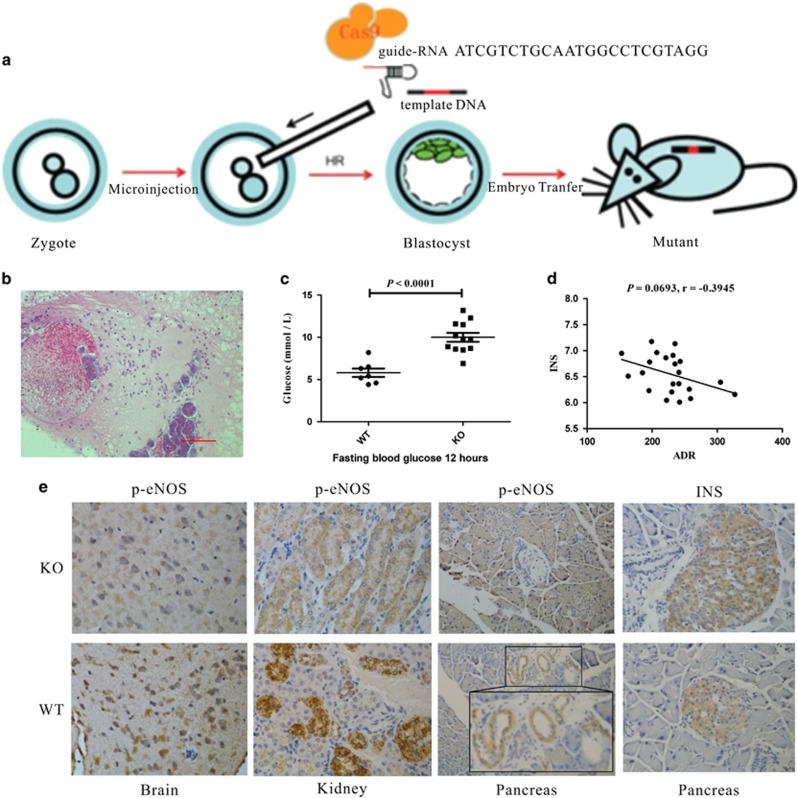
Pathogenesis of fatty pancreas and diabetes in AdrKO mice. (**a**) AdrKO mice for assessing the effect of adropin-deficiency. (**b**) A high number of adipocytes were seen infiltrating the exocrine pancreas of the biopsy from AdrKO mice at the end of 30 weeks on HFD. (**c**)The fasting glucose was significantly higher in AdrKO mice compared to that in WT mice with 8 weeks on HFD. (**d**) Adropin levels were inversely associated with insulin (INS) in AdrHET mice (*n*=22). (**e**) AdrKO mice exhibit reduced eNOS phosphorylation which was reflected as such by brain (neuronal cells), kidney, and pancreas. Islet size appears to be on the larger side and higher expression in AdrKO mice when compared with WT mice

To explore the possibility that adropin serves as an endogenous protective substance for the pancreas, AdrKO mice (Figure 3a) were used to assess the effect of adropin-deficiency on the formation of FP disease and/or diabetes. F5 intercrossed mice were genotyped by Sanger sequencing. Hematoxylin and eosin (H&E) staining of biopsy specimens from AdrKO mice revealed typical histopathological features, including a high number of adipocytes infiltrating the exocrine pancreas (Figure 3b), which is common in human FP disease. We further analyzed glucose levels, which were significantly higher in AdrKO mice (*n*=12, 8.33±1.36 mmol/l, 6.90–13.20 mmol/l) compared with the values of WT mice (*n*=7, 5.80±0.85 mmol/l, 4.40–8.20 mmol/l, *P*<0.0001) (Figure 3c), at 12 months with normal diet. Interestingly, 4 of 12 AdrKO mice developed diabetes (non-fasting blood glucose levels ⩾300 mg/dl). We next explored whether adropin-deficiency is associated with insulin resistance in AdrHET mice. Our results showed that adropin levels were inversely associated with insulin (INS) (*r*=−0.3945, *P*=0.0693, *n*=22) (Figure 3d), as also reflected by INS immunohistochemistry, which showed apparently increased islet size in AdrKO mice compared with WT mice (Figure 3e).

AdrKO mice exhibited reduced eNOS phosphorylation: immunohistochemical staining showed that eNOS phosphorylation at Ser1177 was significantly lower in tissues from AdrKO mice than those of negative control littermates, which was reflected as such in the brain (neuronal cells), kidney (perivascular), and pancreas (perivascular) (Figure 3e).

### Adropin-deficiency is associated with increased severity of obesity-related impaired glucose homeostasis

**Figure 4:**
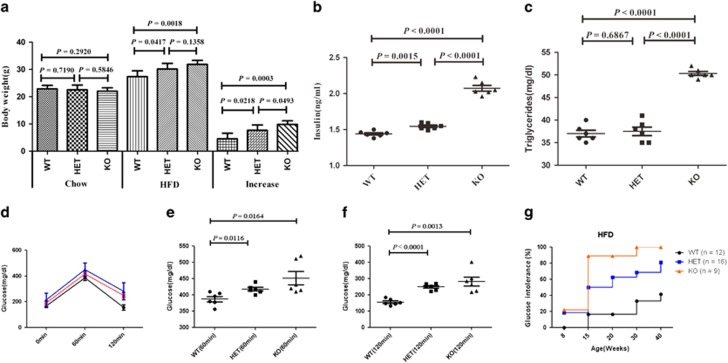
Adropin-deficiency associated with an increased severity of impaired glucose homeostasis associated with obesity. (**a**) The body weight of heterozygous carriers of the null adropin allele (HET) and adropin knockout (KO) mice were significantly higher than that of wild-type control (WT). (**b**) Serum insulin in HET and KO groups were significantly higher than that of WT recorded at the end of 8 weeks on HFD. (**c**) AdrKO mice exhibited fasting hypertriglyceridemia. GTT showed glucose (60 min) (**d**,**e**) and glucose (120 min) (**d**,**f**) were significantly higher than that of WT. (**g**) Almost all of the AdrKO mice developed into diabetes under the high fat induced after 30 weeks

Body weights were not significantly different between the WT, HET and KO groups by pairwise comparison after 8 weeks weaning onto chow (Figure 4a). After 8 weeks on high-fat diet (60% kJ/fat, HFD) (*n*=6/group), body weights of heterozygous carriers of the null adropin allele (HET) and adropin knockout (KO) mice were significantly higher than those of wild-type (WT) controls (*P*=0.0417, *P*=0.0018, respectively); however, there were no significant differences between the HET and KO groups (*P*=0.1358). Serum insulin levels in HET and KO groups were significantly higher than WT values (*P*=0.0015, *P*<0.0001, respectively) at the end of 8 weeks on HFD (Figure 4b). Moreover, AdrKO mice exhibited fasting hypertriglyceridemia (*P*<0.0001 *versus* WT), but AdrHET mice showed no significant difference (*P*=0.6867 *versus* WT) (Figure 4c). The OGTT showed 60-min (Figures 4d and e) and 120-min (Figures 4d and f) glucose levels were significantly higher than WT levels recorded at 8 weeks on HFD. Hyperinsulinemia and hyperglycemia were more severe in adropin knockout mice than in AdrHET mice. Almost all AdrKO mice developed glucose intolerance under high-fat induction at 30 weeks (Figure 4g). Glucose intolerance defined: Fasting plasma glucose is higher than the average value add 3 standard deviation of normal mice, that is fasting plasma glucose >13.9 mmol/l. In one word, impaired glucose tolerance associated with diet-induced obesity was more severe in heterozygous and homozygous carriers of the null adropin allele.

### Expression profiling of pancreatic tissue isolates by RNA-SEQ

**Figure 5:**
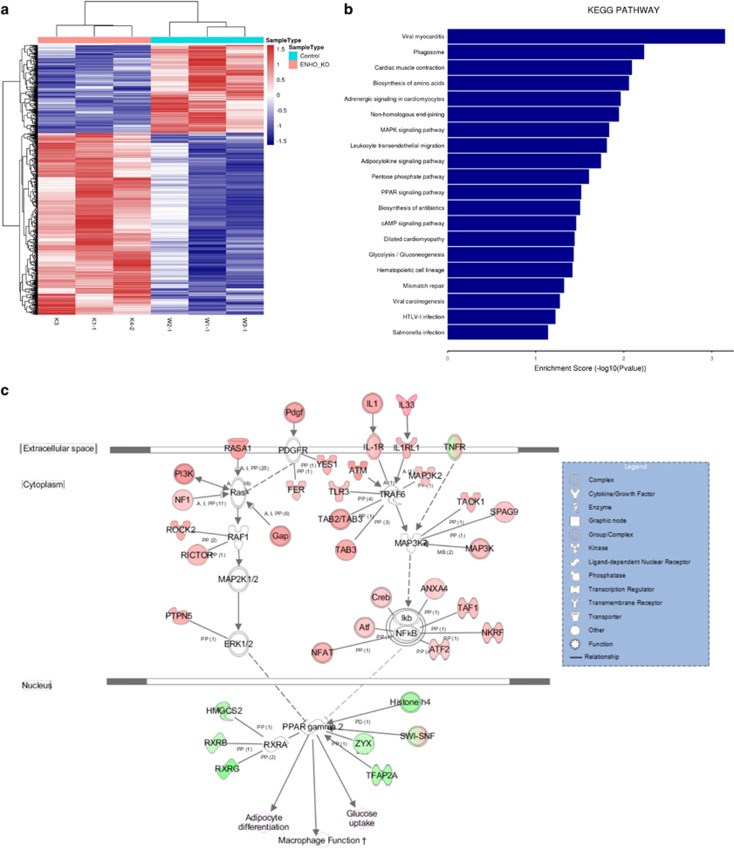
Expression profiling of pancreatic tissue isolates by RNA-SEQ. (**a**) The heatmap depicts hierarchical clustering based on the 973 differentially expressed genes. Unsupervised hierarchical clustering was done with complete linkage. Heatmap visualization for the pancreatic tissues of AdrKO mice and WT mice (*n*=3). Rows: samples; Columns: metabolites; Color key indicates metabolite expression value, blue: lowest; red: highest. (**b**) Importantly KEGG (Kyoto Encyclopedia of Genes and Genomes) pathway mapping of the entire set of differentially expressed genes revealed highly significant molecular interactions for KEGG entries Glycosphingolipid biosynthesis-lacto and neolacto series, Ubiquinone and other terpenoid-quinone biosynthesis. X-axis is an inverse indication of *P*-value or significance. (**c**) IPA signaling pathway analysis of potential intervention targets of adropin-deficiency. Ingenuity analysis of top pathways affected in differentially expressed genes between AdrKO and controls, mRNAs (FDR 10%, FC >1.5). Red symbols specify upregulated expression of genes, whereas green symbols indicate downregulated genes. The color darkness represents the FC intensity

We observed a strong transcriptional interferon response gene signature, and decreased levels of adropin and other interferon-induced cytokines, in pancreatic tissues. A total of 973 putative differentially expressed genes were selected with a cut-off *p*-value of 0.05 and fold change of 1.5 (three biological replicates; Figure 5a). As predicted, most downregulated genes were associated with peroxisome proliferator-activated receptor (PPAR) and adipocytokine signaling pathways; accordingly, ingenuity pathway analysis (IPA) predicted the top upstream regulator to be organism death (*p*-value: 0.00528, Activation z-score: −4.695, Molecules: 77) (Table 1). However, many functions associated with steroidogenesis (downregulation), lipid metabolism (downregulation), and apoptosis (increased) also scored high. Notable upregulated genes included IL1, IL33, and TNFR. The other upregulated genes were mainly observed in AdrKO mice, for example, transcription factor AP-2 epsilon (Tfap2e), heat shock protein 3 (Hspb3), and olfactory receptor 267 (Olfr267). Downregulated genes were mainly enriched in the functions of glycosphingolipid biosynthesis and blood circulation, for example, glucose-6-phosphate dehydrogenase 2 (G6pd2), cAMP responsive element binding protein 3-like 3 (Creb3l3), 5-hydroxytryptamine (serotonin) receptor 1D (Htr1d), and UDP-Gal: beta-GlcNAc beta 1,3-galactosyltransferase, polypeptide 5 (B3galt5). GOSim and SubpathwayMiner were employed for enrichment analysis of coding genes from each specific cluster based on GO terms and KEGG (Kyoto Encyclopedia of Genes and Genomes) pathways. Each cluster was annotated with the enriched functions of the corresponding gene set, such as glycolysis/gluconeogenesis, adipocytokine signaling pathway, and PPAR signaling pathway (Figure 5b) which improves glycemic control, lipid metabolism, and insulin sensitivity in type 2 diabetes.

### Canonical pathway enrichment during adropin deficiency

To determine which pathways were activated during adropin deficiency and potential differences between T2DM and FP, we performed a canonical pathway enrichment analysis using IPA, which showed organismal injury and abnormalities, gastrointestinal disease, and hereditary disorder as the most significantly enriched pathways, as such functions are necessary for gastrointestinal-pancreatic-immunology, confirming the role of adropin deficiency in DM and FP (Supplementary Figure 1). To determine regulatory networks involving significantly up- or downregulated mRNAs in each category, all significant mRNAs (FC >1.5) in each exposure and pathology category were analyzed using an IPA target filter. Adropin deficiency mainly activated the platelet-derived growth factor (PDGF), IL-1, and TNF pathways, and inhibited RXR complex (PPAR˙RXR) formation, thereby inhibiting glucose uptake, adipocyte differentiation, and macrophage function (Figure 5c).

### Adropin-deficiency through the TNF-*α*/NF-kB pathway inhibits PPARG˙RXR complex formation and glycolipid metabolism

**Figure 6:**
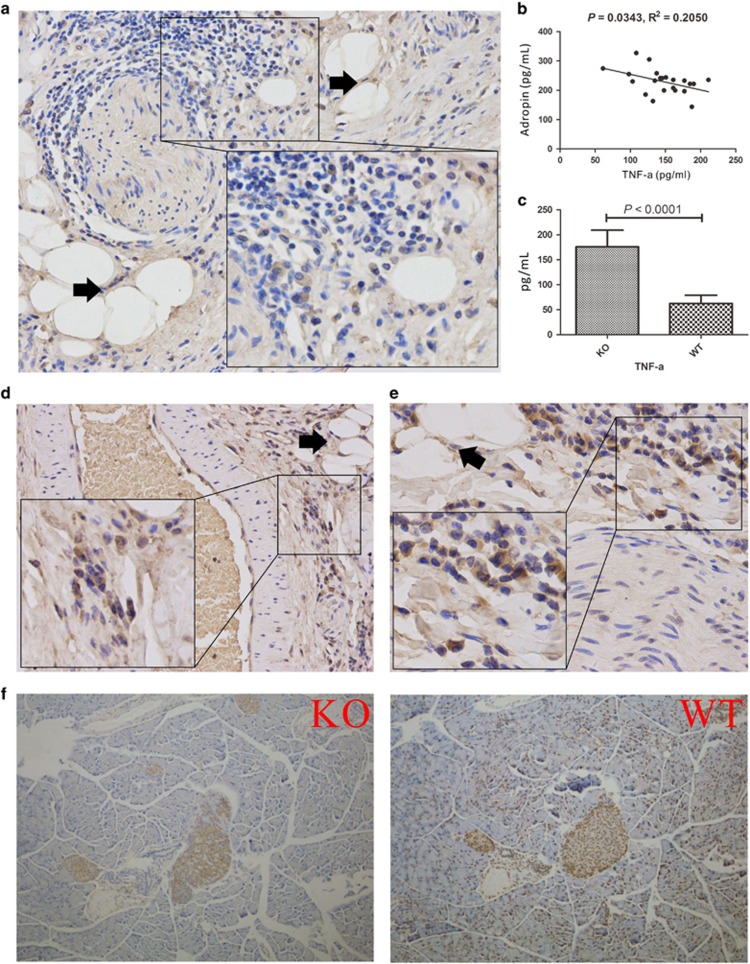
Adropin-deficiency through TNF-*α*/NF-kB pathway to inhibited PPARG. (**a**) TNF-*α* appears to be expressed around the nerve fiber in the pancreas from the FP patients (II7), the black arrow refers to adipose cells. (**b**) Serum TNF-*α* levels were inversely associated with adropin (*R*^2^=−0.2050, *P*=0.0343, *n*=22) in AdrHET mice. (**c**) Serum TNF-*α* was higher in the AdrKO mice than that of WT mice (*P*<0.0001, *n*=3). (**d**) NF-kB was strongly expressed around the small blood vessels and adipose tissue in patient II7, the black arrow refers to adipose cells. (**e**) NF-kB was strongly expressed around the nerve fiber; the black arrow refers to adipose cells. (**f**) PPAR*γ* levels were significantly lower in pancreas from AdrKO mice compared to healthy controls

Meanwhile, pro-inflammatory factors, such as IL-1*β*, TNF-*α* and PDGF, induce cell apoptosis, autophagy, and inhibit PARRG activity. As discussed below, the anti-inflammatory feature of adropin-deficiency seems to positively contribute to mitigate this stress-related inflammatory response. To validate the pathways predicted by RNA-SEQ and IPA, we performed immunohistochemical analysis of pancreatic tissue specimens from a patient (II6) as well as AdrKO and AdrHET mice. Our results showed that serum TNF-*α* levels were inversely associated with adropin (*R*^2^=−0.2050, *P*=0.0343, *n*=22) in AdrHET mice (Figure 6b), while TNF-*α* levels were higher in AdrKO mice than in the WT counterparts (*P*<0.0001, *n*=3) (Figure 6c); this was also reflected by immunohistochemistry, which showed that TNF-*α* appeared to be expressed around adipose tissue in the pancreas specimens from FP patients (Figure 6a). The pro-inflammatory transcription factor nuclear factor kappa B (NF-*κ*B) is a key regulator of inflammation, while the transcription factor peroxisome proliferator-activated receptor gamma (PPAR*γ*) is a key modulator of genes involved in diabetes development. In this study, NF-kB was strongly expressed around nerve fibers (Figure 6d), small blood vessels and adipose tissue (Figure 6e) in patient II6. PPAR*γ* levels were significantly lower in pancreas samples from AdrKO mice compared with normal controls (Figure 6f).

### Adropin deficiency causes reduced eNOS phosphorylation and loss of T_reg_

**Figure 7:**
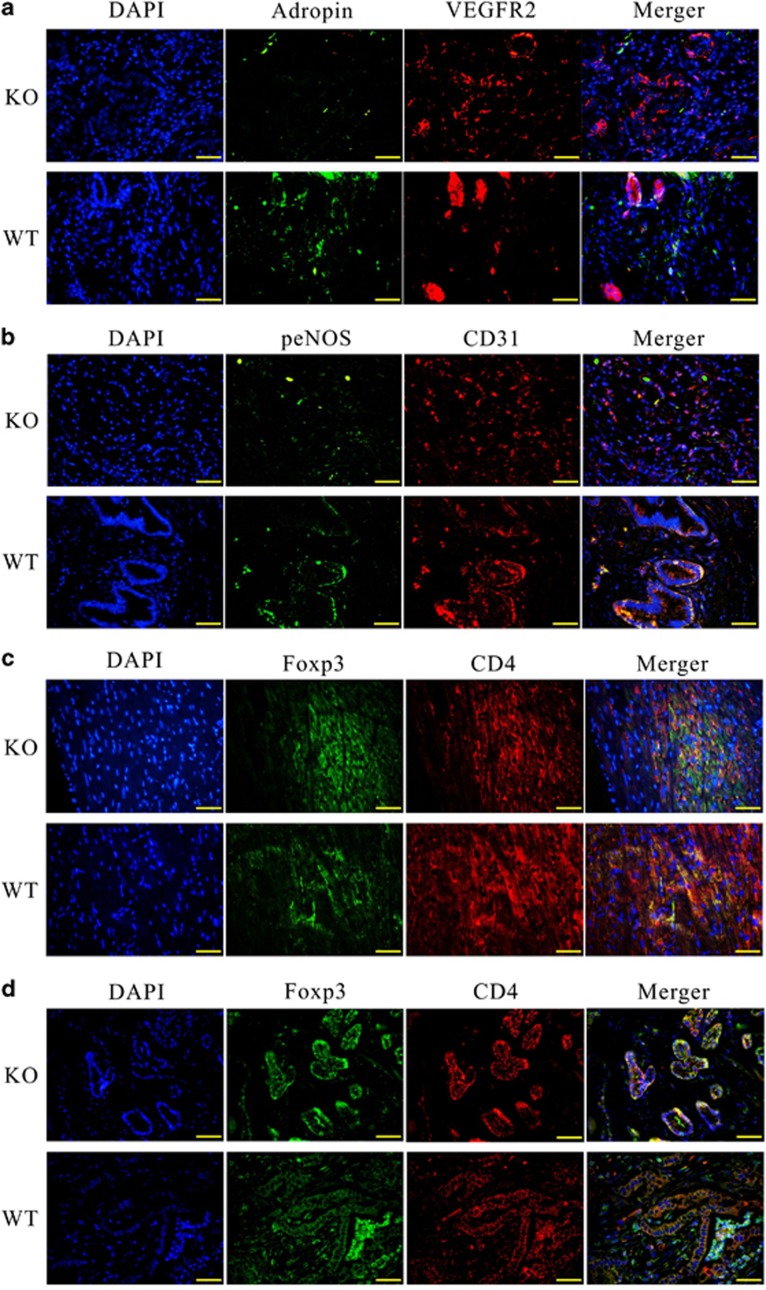
Adropin-deficiency results in loss of p-eNOS and T_reg_ cells. (**a**) Confocal immunofluorescence analysis showing diminutive areas of colocalized DNA in blue, adropin in green and VEGFR2 in red, the overlap of adropin and VEGFR2 (yellow staining in the merged image). (**b**) Confocal immunofluorescence analysis showing small areas of co-localized DNA in blue, CD31 in red and p-eNOS in green, overlap of CD31 and p-eNOS (yellow staining in the merged image) in the endothelial layers. (**c**) Colocalization of DNA (blue), CD4 (red) and Foxp3 (green) indicates T_reg_ cells formation in myocardial. (**d**) Colocalization of DNA (blue), CD4 (red) and Foxp3 (green) indicates T_reg_ cells formation in pancreatic tissues. Original magnification: × 400

Adropin enhances the expression of eNOS in the endothelium via activation of vascular endothelial growth factor receptor 2 (VEGFR2) pathways. Thus, we assessed the co-localization of CD31 (endothelium cell marker), eNOS, adropin, and VEGFR2 in endothelial layers. We found that adropin and p-eNOS levels in pancreatic tissues from AdrKO mice were lower than those obtained for WT mice (Figures 7a and b). For the sub-cellular localization of proteins, tissue immunofluorescence for staining in endothelial layers showed that CD31 and eNOS overlap (yellow staining in the merged image) was also lower in AdrKO mice (Figure 7b), indicating that adropin-deficiency reduced p-eNOS.

Meanwhile, the proportions and absolute amounts of CD4^+^Foxp3^+^ (T_reg_) cells were significantly decreased in myocardial (Figure 7c) and pancreatic tissues (Figure 7d) from AdrKO mice compared with the matched *Enho*^+/+^ littermates, which further suggested that adropin-deficiency was associated with the inhibition of T_reg_. The majority of T_reg_ were distributed only around the pancreatic duct or blood vessels in tissues from AdrKO animals (Figure 7c), and scattered in wild-type specimens (Figure 7d).

## Discussion

Adropin, a recently described peptide hormone produced in the brain, liver, and pancreas, has been reported to have physiologically relevant actions on glucose homeostasis and lipogenesis, exerting significant effects on endothelial function.^19, 20^ It is encoded by the Energy Homeostasis Associated gene (Enho), whose expression is influenced by fasting. However, chronic exposure to high-fat diet is associated with reduced expression of adropin. In the current study, AdrKO mice were sensitive to obesity when fed HFD but not chow. With time, almost all AdrKO mice developed diabetes under high-fat induction. Furthermore, there was a significant inverse correlation between adropin and relative T_reg_ amounts in patients with FP and T2DM. *In vivo*, adropin-deficient mice displayed loss or abnormal distribution of T_reg_.

It has been reported that increased triglyceride and FFA levels causes ectopic fat deposition in the liver, heart, muscles, and pancreas, a term referred to as steatosis.^1, 6^ Surprisingly, FP was not correlated with increased cholesterol, glyceride, or FFA amounts in this study. Particularly, there was a clear phenomenon in a three-generation family of Chinese origin with the common feature of diabetes attacks and FP disease, and all the affected members showed adropin deficiency, which led to the question whether the latter is associated with Enho mutations. Excitingly, there were p.Cys56Trp heterozygous protein-altering variants and p.Tyr72Tyr synonymous mutations in many members of this family across three generations as well as other unrelated patients with FP and T2DM. p.Tyr72Tyr synonymous mutations were located at the predicted tyrosine phosphorylation site, which may affect mRNA folding, thereby perturbing the translation process^21^; alternatively, Enho was mainly regulated by miRNAs (Supplementary Figure 2). Meanwhile, *Enho* mutations and adropin-deficiency were found in patients with FP and T2DM, which revealed the two diseases share similar pathogenetic mechanisms and multiple metabolic derangements which can accelerate the development and progression of both conditions. To gain more insights into genotype–phenotype correlations in adropin-deficiency, we used AdrKO mice to investigate genotype–proteotype–phenotype correlations in FP and T2DM patients with adropin-deficiency. In this study, homo- and heterozygous carriers of the null adropin allele exhibited increased severity of impaired glucose homeostasis and fat metabolism disorder compared with wild-type mice. *In vivo*, Enho^−/−^ mice showed FP disease and diabetes, with a high number of adipocytes infiltrating within the exocrine pancreas.

Elevated blood levels of glucose and insulin were detected in AdrKO and AdrHET mice. Furthermore, this study described the RNA-seq profiles of pancreatic tissues from AdrKO mice; there were 86 genes identified as involved in glycosphingolipid or ubiquinone biosynthesis, adipocytokine signaling pathway, PPAR signaling pathway, and the biosynthesis of other terpenoid-quinones. PPARs are lipid-activated transcription factors; their characterized target genes encode proteins that participate in lipid homeostasis.^22, 23^ In this study, RXRs had lower expression levels in AdrKO mice; meanwhile, coordination and cross talk among multiple components of this network are critical to ensure correct energy balance and insulin resistance. Other examples are *N*-acetyltransferase,^24, 25^ glucose-6-phosphate dehydrogenase (G6PD), transcription factor AP-2, and heat shock protein, which contribute to susceptibility to type 2 diabetes and FP disease by inhibiting glucose-induced insulin secretion in pancreatic *β* cells or naringin attenuated insulin resistance^26^; adropin deficiency downregulated peNOS whose uncoupling contributes to endothelial dysfunction.^13, 27^ To further explore the signal transduction pathways involved in adropin-attenuated impaired angiogenesis in diabetic mice, we examined the effects of adropin on eNOS phosphorylation in AdrKO mice, which showed reduced eNOS (Ser1177) phosphorylation within perivascular cells from the kidney and pancreas, as well as neuronal cells.

T_reg_ are paramount to the initiation and propagation of metabolic-inflammation, with adipose tissue acting as the initial site of obesity-induced inflammation. Adipose tissue expansion can occur in two ways, with hyperplasia or hypertrophy, that is, increases in adipocyte number and size, respectively. Hypertrophic obesity is associated with size expansion of existing adipocytes, with the morphology showing greater adipocyte volume. Hypertrophic obesity is usually associated with insulin resistance, and the hyperplasic type with insulin sensitivity. Adipocytes increase in number and are therefore better equipped to deal with the demand for excess energy/lipid storage.^28, 29^

Adropin-induced metabolic-inflammation in metabolic and immune cells of the adipose, liver, pancreas and skeletal muscle contributes to the development of obesity-induced insulin resistance.^30^ The present study showed adropin-deficiency could inhibit insulin signaling and glucose transport. *In vivo,* AdrKO and AdrHET mice showed increased fasting glucose, insulin, and TG levels. Even more compelling are glucose tolerance test (GTT) data showing that loss of Enho results in an insulin resistance phenotype. Under the control of adropin, combined inflammation and diet can determine the metabolic pathway utilized by the cell. Inflammation undergoes metabolic reprogramming, with a switch from the energy-efficient oxidative phosphorylation to glycolysis, a less energy efficient option; downstream metabolites can then engage in feedback inhibition, and cause further inflammation and oxidative stress. In addition, autophagy is dynamically regulated in T_reg_, whose specific loss results in increased apoptosis and impaired lineage stability^31, 32^; this may lead to apoptosis of normal pancreatic cells, and lipid spillover from the expanding adipose tissue ends up causing fat replacement or infiltration within the pancreas, which is different from the mechanism of nonalcoholic fatty liver formation. This may be the reason for most FP patients having normal blood lipid levels.^33, 34, 35^

The effects of pancreatic fat on insulin resistance and beta-cell function have been investigated in animal and human studies.^36, 37^ However, it remains unclear whether FP disease and type 2 diabetes share common mechanisms. In this study, we demonstrated that diabetes and pancreatic fat disease mainly result from genetic susceptibility and diet interactions. A thorough understanding of adropin’s actions would advocate for the use of this protein for therapeutic purposes in diabetes and/or FP disease. We deduce that Enho mutations as well as lifelong sugar carbohydrate and fat-induced adropin deficiency may provide additional damage to the pancreas in fat accumulation and T2DM, by altering the number or function of T_reg_ and stimulating autophagy.

## Materials and methods

### Study population

A three-generation family of Chinese origin with the common feature of diabetes attacks (Figure 1a) included three patients with FP, 18 T2DM cases, and 13 normal controls. Additional 19 unrelated patients with FP and 58 T2DM cases were included; 220 population-matched healthy individuals served as controls. FP was diagnosed with increased echogenicity of the pancreatic body over that of the kidney based on the pathological diagnosis. This study was approved by the Ethics Committee of Fujian Medical University.

### Analysis of *Enho* mutations, adropin, and the relative T_reg_ cells number

Blood was collected and DNA extracted using a Tiangen Genomic extraction kit (Beijing, China). Full-length *Enho* was amplified, purified, and sequenced. Serum levels of adropin from the patients with FP or T2DM and normal controls were measured using a specific enzyme-linked immunosorbent assay (ELISA) kit (R&D Systems, Minneapolis, MN, USA). We quantitated the relative T_reg_ cells number by analyzing CD4^+^CD25^+^FOXP3^+^ cells with the flow cytometric assay.

### Gene targeting in AdrKO mice

AdrKO mice were generated by clustering regularly interspaced short palindromic repeats (CRISPR)-Cas9 by the Shanghai Biomodel Organism Science & Technology Development Co., Ltd., in the C57BL/6J background (Figure 2a). AdrKO, AdrHET, and wild-type (WT) mice were housed under a 12 h/12 h light–dark cycle at constant temperature (23±1 °C) with free access to water. The animals were maintained on chow diet (Chow; 60% kJ provided by carbohydrates; 26% kJ/protein, and 14% kJ/fat) or HFD (60% kJ/fat, 20% kJ/carbohydrate, and 20% kJ/protein).

### Glucose, insulin, and serum lipid measurements

For the GTT, mice were fasted overnight and injected D-glucose (1–2 g/kg body weight). Blood samples were obtained at various time points (0, 60 min, and 120 min) from the various types of mice (AdrKO, AdrHET, and WT), by tail-vein nick. Insulin levels in plasma were measured with an ELISA kit (Crystal Chem, Downers Grove, IL, USA). Serum lipids (triglycerides, cholesterol, and HDL) were measured by IDEXX Laboratories (West Sacramento, CA, USA).

### Histology and immunohistochemistry

Pancreas tissue were fixed in 4% formalin overnight, embedded in paraffin, sectioned at 4 mm and stained with H&E for pathology. The following antibodies were used: anti-Insulin (ABclonal), anti-p-eNOS (Ser1177) (Santa Cruz, Santa Cruz, CA, USA).

### RNA-seq and pathway mapping analysis

Assignment of metabolites was identified based on the published literature and databases such as HMDB, KEGG, PubChem compound database and SMPDB.^13^ Subsequent pathway mapping analysis was conducted with the IPA metabolomics model (http://www.Ingenuity.com/products/ pathways_analysis.html).

### Localization of VEGFR2/adropin, CD31/p-eNOS and CD4/FOXP3

Immunofluorescence confocal microscopy was also undertaken to determine the correlation of VEGFR2 and adropin. VEGFR2 was detected with rabbit anti-human antibody (Sangon, Shanghai, China) and labeled with a goat anti-rabbit secondary antibody conjugated either to Cy3. Adropin was detected with a mouse anti-human antibody (Sanying, Wuhan, China) and labeled with a goat anti-mouse secondary antibody conjugated to FITC. Nuclei were costained using 4',6-diamidine-2- phenylidole dihydrochloride (DAPI).

EC was identified by staining using antiCD31 which was detected with a rabbit antihuman antibody (Santa, Santa Cruz, CA, USA) and labeled with a goat anti-rabbit secondary antibody conjugated to Cy3. p-eNOS was detected with a mouse anti-human antibody (Santa, USA) and labeled with a goat anti-mouse secondary antibody conjugated to FITC. And nuclei were costained using DAPI.

T_reg_ was identified by staining using antiCD4 and antiFoxp3. CD4 was detected with a rabbit antihuman antibody (Sanying, Wuhan, China) and labeled with a goat anti-rabbit secondary antibody conjugated to Cy3. Foxp3 was detected with a mouse anti-human antibody (Santa, USA) and labeled with a goat anti-mouse secondary antibody conjugated to FITC. Nuclei were costained using DAPI.

### Statistics

Statistical differences between groups were assessed by the nonparametric Mann–Whitney *U*-test for two groups and Kruskal–Wallis test for more than two groups. Bonferroni–Dunn’s correction method was applied for *post hoc* multiple pair-wise comparisons. Spearman’s rank correlation coefficient estimated the degree of association between two variables. Significance was calculated at *P*<0.05 by GraphPad Prism 5 (La Jolla, CA, USA).

## Figures and Tables

**Table 1:** Disease and Functions

**Function annotation**	***P*****-value**	**Activation z-score**	**Molecules**
Lymphoid cancer and tumors	1.57E-03	−2.246	100
Lymphohematopoietic neoplasia	1.39E-03	−2.274	105
Hematological neoplasia	1.78E-03	−2.595	104
Lymphoproliferative malignancy	1.89E-03	−2.944	97
Lymphoid cancer	2.10E-03	−2.578	98
Lymphohematopoietic cancer	3.28E-03	−2.595	101
Hematologic cancer	4.04E-03	−2.941	100
Abdominal neoplasm	9.26E-10	−2.143	304
Gonadogenesis	1.42E-03	2.289	23
Gametogenesis	4.76E-04	2.254	21
Hypoplasia of lymphoid organ	1.04E-02	−2.216	8
Agenesis	3.39E-03	−2.138	8
DNA replication	2.59E-03	2.415	12
Radiosensitivity of cells	8.85E-05	−2.236	5
Incidence of lymphoma	1.41E-02	−2.428	6
Differentiation of blood cells	9.83E-03	2.049	30
Spermatogenesis	4.45E-04	2.066	19
Development of genital organ	1.47E-03	2.289	24
Organismal death	5.28E-03	−4.695	77
