# Supplementary figures and images for: Adropin deficiency worsens HFD-induced metabolic defects
Source: Cell Death Dis. 2017 Aug 24;8(8):e3008–. doi: 10.1038/cddis.2017.362 (PMC5596552; doi:10.1038/cddis.2017.362)

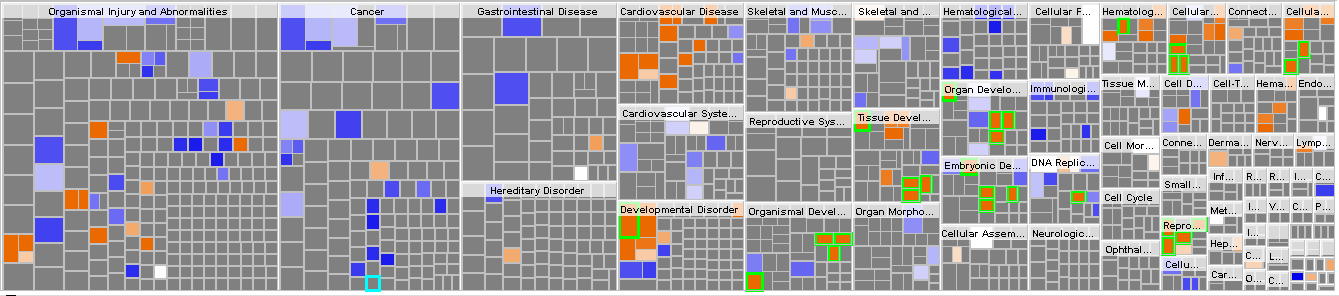

Supplement: Supplementary Figure 1 [file cddis2017362x1.png]

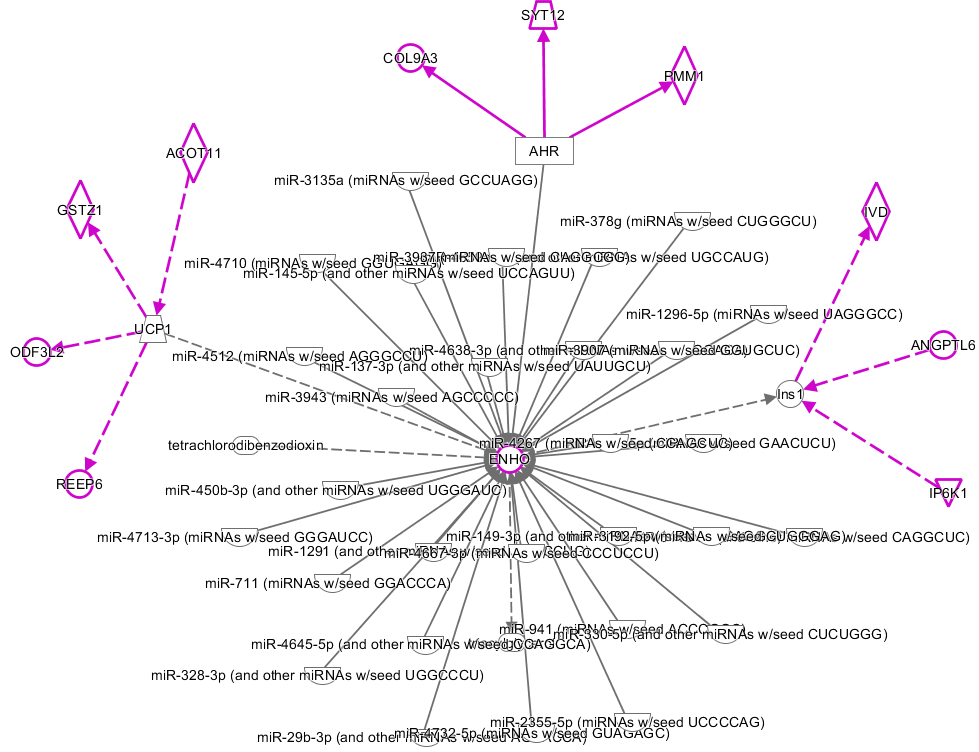

Supplement: Supplementary Figure 2 [file cddis2017362x2.png]
