# Supplementary material for: Adropin deficiency worsens HFD-induced metabolic defects
Source: Cell Death Dis. 2017 Aug 24;8(8):e3008–. doi: 10.1038/cddis.2017.362 (PMC5596552; doi:10.1038/cddis.2017.362)
Supplement: Supplementary Figure Legends [file cddis2017362x3.docx]

**Supplementary Figure Legends**

**Supplementary Fig.1:** Disease & Function.

**Supplementary Fig.2:** Protein-protein interaction network of ENHO.
